# Chemoresistance-Associated Silencing of miR-4454 Promotes Colorectal Cancer Aggression through the *GNL3L* and *NF-κB* Pathway

**DOI:** 10.3390/cancers12051231

**Published:** 2020-05-14

**Authors:** Thetchinamoorthy Kannathasan, Wei-Wen Kuo, Ming-Cheng Chen, Vijaya Padma Viswanadha, Chia-Yao Shen, Chuan-Chou Tu, Yu-Lan Yeh, Mahalakshmi Bharath, Marthandam Asokan Shibu, Chih-Yang Huang

**Affiliations:** 1 Graduate Institute of Biomedical Sciences, China Medical University, Taichung 404, Taiwan; kannathasan45@gmail.com; 2 Department of Biological Science and Technology, China Medical University, Taichung 404, Taiwan; wwkuo@mail.cmu.edu.tw; 3 Department of Surgery, Division of Colorectal Surgery, Taichung Veterans General Hospital, Taichung 407, Taiwan; claudiochen7@gmail.com; 4 Faculty of Medicine, National Yang-Ming University, Taipei 112, Taiwan; 5 Department of Biotechnology, Bharathiar University, Coimbatore 641046, India; padma.vijaya@gmail.com; 6 Department of Nursing, Meiho University, Pingtung 912, Taiwan; x00003061@meiho.edu.tw; 7 Division of Chest Medicine, Department of Internal Medicine, Armed Force Taichung General Hospital, Taichung 404, Taiwan; tu4697@gmail.com; 8 Department of Pathology, Changhua Christian Hospital, Changhua 500, Taiwan; 1867@cch.org.tw; 9 Department of Medical Technology, Jen-The Junior College of Medicine, Nursing and Management, Miaoli 35664, Taiwan; 10 Institute of Research and Development, Duy Tan University, DA Nang 550000, Vietnam; mahalakshmibharath05@gmail.com; 11 Holistic Education Center, Tzu Chi University of Science and Technology, Hualian 970, Taiwan; Shibu.m.a@gmail.com; 12 Cardiovascular and Mitochondrial Related Disease Research Center, Hualian Tzu Chi Hospital, Buddhist Tzu Chi Medical Foundation, Hualian 970, Taiwan; 13 Department of Medical Research, China Medical University Hospital, China Medical University, Taichung 404, Taiwan; 14 Department of Biotechnology, Asia University, Taichung 413, Taiwan

**Keywords:** colorectal cancer (CRC), irinotecan (CPT-11), miR-4454, *GNL3L* (guanine nucleotide-binding protein-like-3-like)

## Abstract

Guanine nucleotide-binding protein-like-3-like (*GNL3L*) is a crucial regulator of *NF-κB* signaling that is aberrantly activated during diverse chemoresistance-associated cellular processes. However, the molecular mechanisms of *GNL3L* tumor initiation and resistant state are largely unknown. Moreover, the identification of predictive biomarkers is necessary to effectively generate therapeutic strategies for metastatic human colorectal cancer (CRC). This study aims to identify how cells acquire resistance to anticancer drugs and whether the downregulation of miR-4454 is associated with the progression of CRC. Here, we have shown that the overexpression of miR-4454 in resistant tumors is a crucial precursor for the posttranscriptional repression of *GNL3L* in human chemoresistant CRC progression, and we used doxycycline induced miR-4454 overexpression that significantly reduced tumor volume in a subcutaneous injection nude mice model. Together, these observations highlight that the downregulation of miR-4454 in resistant clones is prominently responsible for maintaining their resistance against anticancer drug therapy. Our study indicates that the development of miR-4454 as a microRNA-based therapeutic approach to silence *GNL3L* may remarkably reduce oncogenic cell survival that depends on *GNL3L/NF-κB* signaling, making miR-4454 a candidate for treating metastatic human CRC.

## 1. Introduction

Colorectal cancer (CRC) is the third most common type of malignant disease in men and women, and according to a recent statistic, there are an estimated 140,250 new cases of CRC diagnosed in the United States alone [1]. Although various therapeutic strategies have been developed, the five-year survival rate for patients with metastatic CRC remains low (around 13.5%).

Drug resistance in CRC is a crucial challenge in the treatment of metastatic cancer. Recently, numerous mechanisms have been identified to be responsible for the development of resistance to first-line chemotherapeutic drugs. The initial response to the first-line chemotherapy drug may vary as tumor cells reemerge at a relatively high frequency during relapse in a sensitive population after subsequent treatment failures with various anticancer drugs [2,3,4,5,6,7,8]. Drug resistance is widely observed in various cancers because of their ability to survive through crosstalk with factors in multiple signaling pathways [9,10,11]. Thus, the identification of predictive biomarkers is necessary to effectively generate therapeutic strategies for metastatic human CRC.

MicroRNAs are small noncoding RNAs that can influence chemoresistance through the epigenetic regulation of various cancer cell phenotypic states, such as proliferation, metastasis, cancer cell stemness, cell cycle control, and cell death [12,13,14].

LoVo cells, a colon cancer cell line originally isolated from a metastatic tumor nodule in the left supraclavicular region of a 56-year-old Caucasian male patient, have been histologically proven as adenocarcinoma stage IV colon cancer cells that had spread to nearby lymph nodes and other organs or tissues (liver and lungs) [15]. Previous studies on irinotecan-resistant (CPT-11-R) cell lines showed that the activation of the *EGFR/IKKα/β/NF-κB* pathway leads to enhanced metastasis [10].

Guanine nucleotide-binding protein-like-3-like (*GNL3L*) is a putative nucleolar GTPase belonging to the HSR1-MMR1 family and is conserved among eukaryotes. *GNL3L* has an N-terminal basic domain and a central guanosine triphosphate (GTP)-binding domain. GTP-binding motifs also play an important role in the nuclear localization of *GNL3L* [16]. *GNL3L* interacts with mouse double-minute 2 homolog to prevent ubiquitination as well as with telomere repeat binding factor 1 [17]. Recently, *GNL3L* has been identified as one of the factors responsible for the maintenance of the tumorigenic properties of tumor-initiating cells, and it promotes NF-κB-mediated cell survival via the upregulation of antiapoptosis-related genes [18,19].

This study aimed to identify how cells acquire resistance to anticancer drugs and whether the downregulation of miR-4454 is associated with the progression of CRC. Here, we generated an irinotecan (CPT-11) drug-resistant clone (CPT-11-R) from the LoVo cell line by stepwise increments of CPT-11 drug exposure during culturing. Then, we found the microRNAs that were differentially expressed in CPT-11-R-resistant clones with respect to LoVo cells and identified the upregulated and downregulated microRNAs. Furthermore, we have identified miR-4454 dysregulation and secretion through extracellular vehicles (EVs) in resistant cells. We found that most resistant cells significantly downregulated miR-4454 to regulate the *GNL3L* gene and thus induce the drug-resistant state. We discovered that miR-4454 directly targets *GNL3L* and reduces tumorigenicity. In addition, we found that, as a consequence of miR-4454 overexpression, the CPT-11-R clones had increased rates of apoptosis and G2/M arrest when treated with the first-line CPT-11 drug, and we also observed that the inhibition of miR-4454 in LoVo cells was inversely correlated with miR-4454-overexpressing CPT-11-R cells. Our study indicates that the development of miR-4454 as a microRNA-based therapeutic approach for silencing *GNL3L* may remarkably reduce oncogenic cell survival that depends on *GNL3L/NF-κB* signaling, making miR-4454 a candidate modality for treating metastatic human CRC.

## 2. Results

### 2.1. Generation of Drug-Resistant Cell Lines

**Figure 1:**
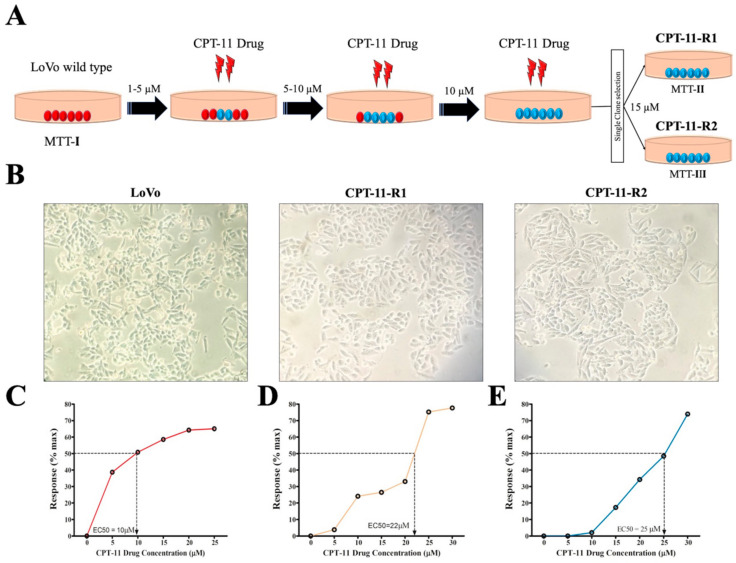
Generation of drug-resistant cell lines. A drug-resistant stable cell line developed from the LoVo cell line. (**A**) Drug-resistant stable cell line generation through stepwise challenge using CPT-11 (irinotecan) in the LoVo colon cancer cells for 6 months. (**B**) Morphology changes in the LoVo cells and CPT-11-R clones (20×). Drug response was assessed using cell proliferation (MTT) assay. (**C**) Treatment with 10 µM CPT-11 drug generated EC_50_ values in the LoVo cell line at 48 h. (**D**,**E**) 25 µM CPT-11 drug treatment to reach the EC_50_ value in CPT-11-R-resistant clones at 72 h. The results are presented as mean ± standard error of the mean (*n* = 3).

Drug-resistant cell lines were generated by plating 10^6^ cells in 10 cm plates, and thereafter treated with 1 µM CPT-11 drug for 12 days. The medium was replaced every 72 h with fresh medium containing the drug. Following the same procedure, the cells were challenged with 1 µM to 10 µM CPT-11 drug to continue enhancing the drug resistance for six months (Figure 1A). Then, we compared the morphological changes of the LoVo cells and the CPT-11-R clones (Figure 1B). The level of drug resistance was determined using 3-2,5-diphenyltetrazolium bromide (MTT) assay. First, we determined the LoVo cell EC_50_ values after CPT-11 drug treatment at different concentrations (Figure 1C). After the generation of a single clone with resistance to 10 µM CPT-11, an isolation method was used to isolate the different clones, and then, we performed another MTT assay to determine the resistant cell drug response (% maximum). Finally, a single clone was selected and maintained with 15 µM CPT-11 for one month, and the resistant cell drug response was determined once again using MTT (Figure 1D,E).

### 2.2. Apoptotic Reduction and Cell Cycle Alteration Increase CPT-11-R Cell Chemoresistance

**Figure 2:**
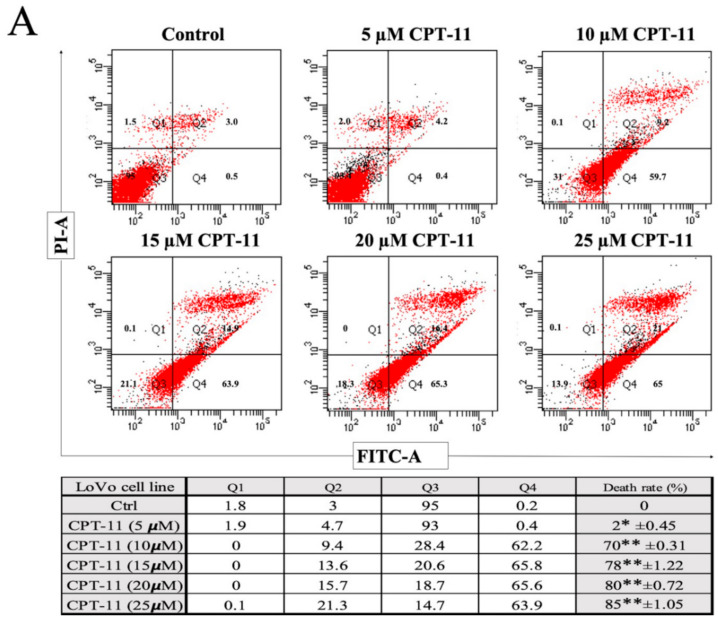

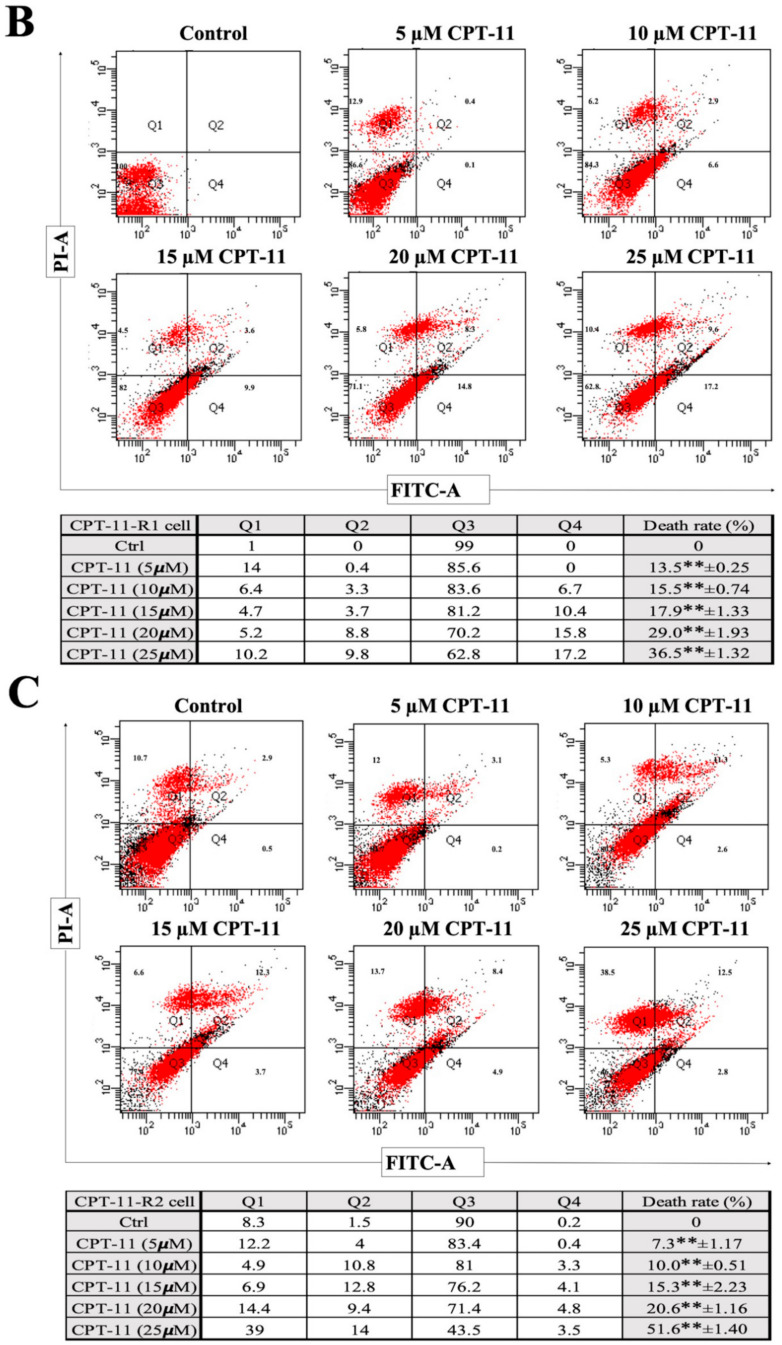

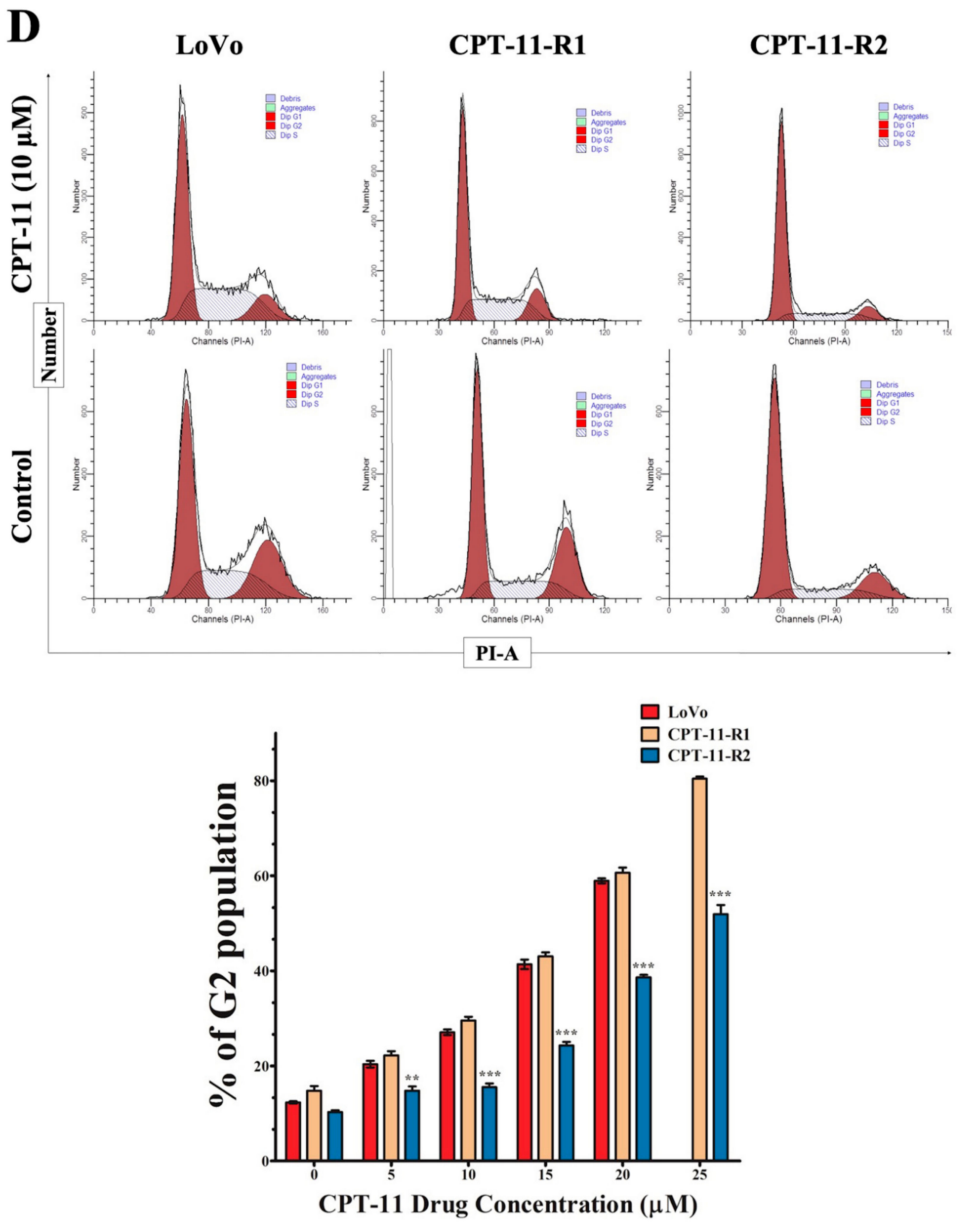
Reduced apoptosis and cell cycle alterations in chemoresistant CPT-11-R cell clones. (**A**) Apoptosis for LoVo (Table shows cell death rate in %), (**B**) apoptosis for CPT-11-R1 (Table shows cell death rate in %), (**C**) apoptosis for CPT-11-R2 (Table shows cell death rate in %). (**D**) The cells were treated with different concentrations of CPT-11 drug (5, 10, 15, 20, 25 µM) for 48 h (LoVo) and 72 h (resistant clones). Cell death rate and cell cycle determined using the FACS analysis. The results are presented as mean ± standard error of the mean (*n* = 3). * *p* < 0.05, ** *p* < 0.01, *** *p* < 0.001 compared to control.

To determine whether the inhibition of the cell death rate had an impact on the observed drug resistance of the LoVo cells and CPT-11-R clones, we performed a flow cytometry-based Annexin V/propidium iodide (PI) cell death assay on the LoVo cells and the CPT-11-R clones. Consistent with the MTT results, the Annexin V/PI cell death assay demonstrated that CPT-11 drug treatment for 48 h increased the cell death rate in a dose-dependent manner in the LoVo cells (Figure 2A), and the CPT-11-R clone cell death rate was lower than that of the LoVo cells at 72 h (Figure 2B,C). The percentage of the apoptotic cells among the nontreated cells was nearly the same as that of the LoVo and CPT-11-R cells.

To investigate the alteration of the cell cycle regulation that is related to the enhanced chemoresistance to the CPT-11 drug, we analyzed the cell cycle points for the LoVo cells and the CPT-11-R clones. We used different concentrations of the CPT-11 drug (5, 10, 15, 20, 25 µM) to treat the LoVo and CPT-11-R cells. Previous studies have pointed out that the CPT-11 drug (irinotecan) blocks the cell cycle in the G2/M phase. Consistent with these studies, we found that the LoVo cells were arrested in the G2/M phase of the cell cycle in a CPT-11 concentration-dependent manner (Figure 2D, red bar chart). Interestingly, we found that, compared with the percentage of the control cells, ~70% of the CPT-11-R cells were in the G0/G1 phase. The cells were treated with different concentrations of CPT-11 (5, 10, 15, 20, 25 µM), and those treated with a drug concentration of 25 µM were arrested in the G2/M phase (Figure 2D, brown, blue bar chart). Taken together, our results show that the LoVo cells treated with different concentrations of the CPT-11 drug were arrested in the G2/M phase in a concentration-dependent manner at 48 h. However, the cell cycle arrest of the CPT-11-treated CPT-11-R cells was delayed because there were more cells accumulated in the G0/G1 phase than were arrested in the G2/M phase when we applied higher concentrations of the drug. Taking into consideration the fact that clone-2 represented stronger drug resistance as determined by their low susceptibility to apoptosis and better G2/M phase protection against CPT-11 drug, we proceeded with using clone-2 for further studies.

### 2.3. CPT-11-R Cells Show Enhanced First-Line Chemoresistance, Colony Formation, Invasion, and Migration Ability

**Figure 3:**
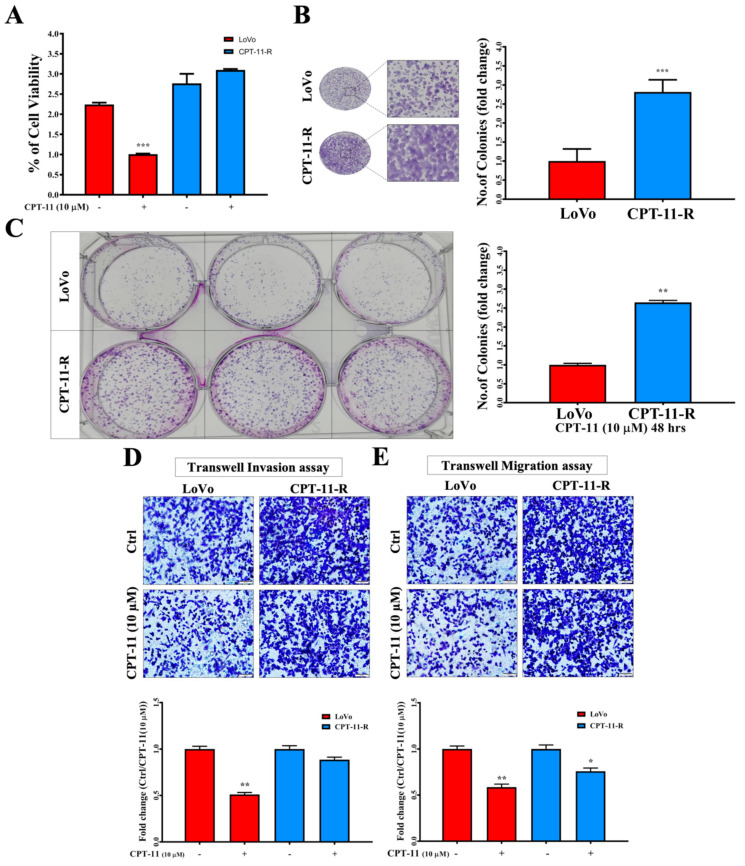
CPT-11-R cells demonstrate enhanced first-line chemoresistance, colony formation, and invasion and migration ability. (**A**) The chemoresistance ability of CPT-11-R cells was determined after 10 µM CPT-11 drug treatment for 48 h in comparison with LoVo cells. (**B**) The colony formation ability of the CPT-11-R cells was determined without treatment under normal conditions and fold change normalized with untreated control group. (**C**) CPT-11-R cell colony formation ability with 10 µM CPT-11 treatment. (**D**) Invasion and (**E**) migration assay (scale bar: 100 µm). The results are presented as mean ± standard error of the mean (*n* = 3). * *p* < 0.05, ** *p* < 0.01, *** *p* < 0.001 compared to control.

We investigated the percentage of the viable LoVo and CPT-11-R cells after treatment with 10 µM CPT-11 for 48 h. Treatment with 10 µM CPT-11 significantly reduced the cell viability by 50% in the LoVo cells compared with that of the CPT-11-R cells. The CPT-11-R cells showed no significant reduction compared to the LoVo and CPT-11-R control cells (Figure 3A). Next, we determined the colony formation ability of the LoVo and CPT-11-R cells. When the cells were cultured under normal conditions and with the CPT-11 drug, the resistant cells showed a significant difference in colony formation ability compared to the LoVo cells (Figure 3B,C). To examine the invasion and migration ability of the LoVo and CPT-11-R cells, we used a transwell assay and found that the CPT-11-R cells exhibited significantly increased cell invasion and migration ability compared with the LoVo cells (Figure 3D,E). Further, we analyzed the molecular intricacies in the LoVo and CPT-11-R cells with a special convergence on the *NF-κB* pathway, since the aberrant activation of the *NF-κB* pathway plays key roles in numerous oncogenic processes [10].

### 2.4. Downregulation of miR-4454 Is Attributed to Elevated Levels of GNL3L in the CPT-11-R Cells

**Figure 4:**
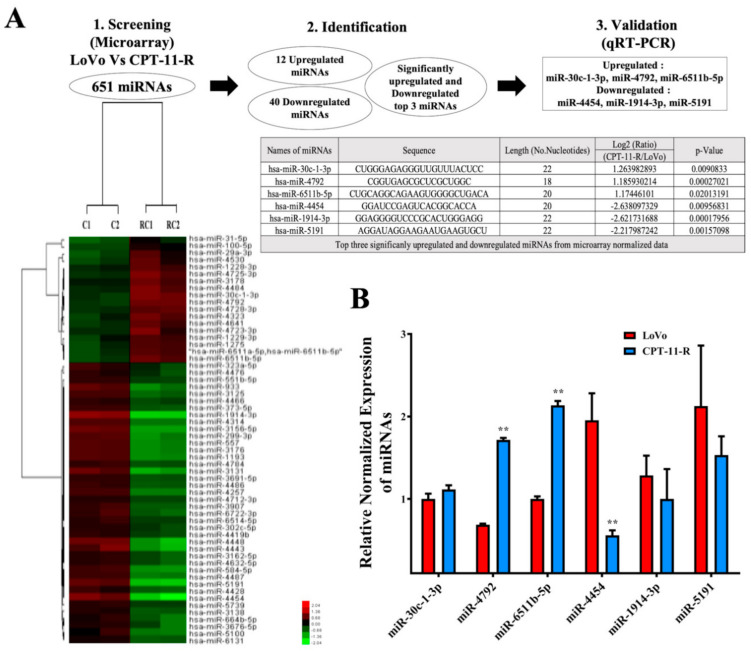

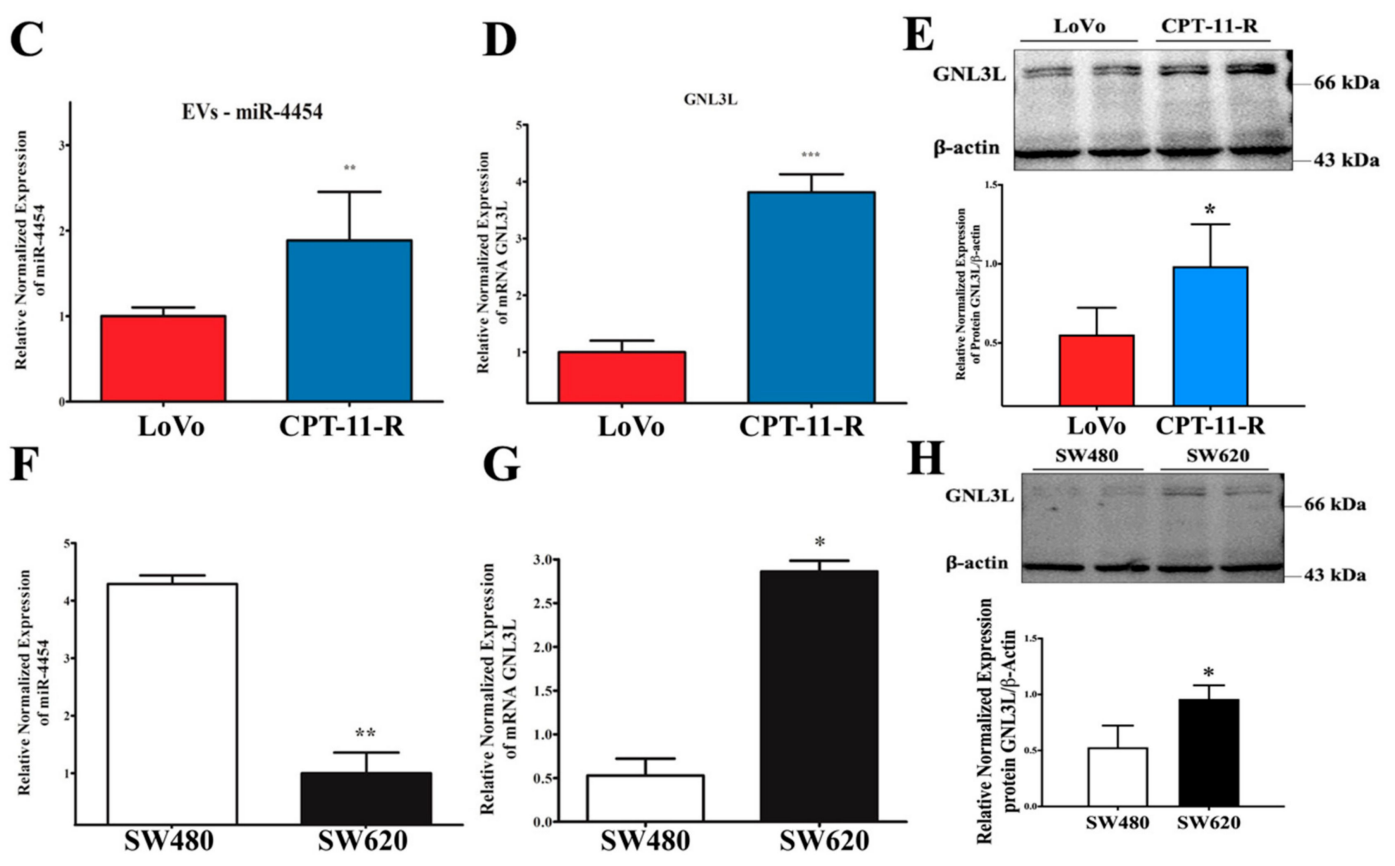
The downregulation of miR-4454 is attributed to the overexpression of *GNL3L* in the CPT-11-R cells. (**A**) Microarray data for LoVo versus CPT-11-R cell line differential expression. (**B**) qRT-PCR for LoVo and CPT-11-R cells to reevaluate the miR-4454 expression level. (**C**) The miR-4454 expression level in the extracellular vehicle. (**D**) Results of qRT-PCR showing LoVo and CPT-11-R cell *GNL3L* expression levels. (**E**) *GNL3L* protein level and (**F**) miR-4454 expression level in the microsatellite stable primary SW480 versus metastatic SW620 cell line. (**G**) SW480 versus SW620 *GNL3L* mRNA expression level and (**H**) *GNL3L* protein expression levels. The results are presented as mean ± standard error of the mean (*n* = 3). *p < 0.05, ** *p* < 0.01, *** *p* < 0.001 compared to control.

We explored the aberrant expression of microRNAs in the LoVo and CPT-11-R cells using a microarray technique (Figure 4A). Then, we reevaluated the aberrant microRNA expression levels in the resistant cells (upregulated miR-30c-1-3p, miR-4792, and miR-6511b-5p and downregulated miR-4454, miR-1914-3p, and miR-5191) using quantitative real time-polymerase chain reaction (qRT-PCR), and the result demonstrated that miR-4454 was significantly downregulated compared with the LoVo cells (Figure 4B). We also evaluated whether the LoVo and CPT-11-R cell culture media had EVs that were releasing miR-4454. qRT-PCR results showed that higher levels of miR-4454 were detected in the EVs of CPT-11-R cells than in the EVs of LoVo cells (Figure 4C). *GNL3L* is a potential target of miR-4454 according to predictions of the TarBase and TargetScan 7.1 online tool. We identified the *GNL3L* mRNA expression level using qRT-PCR to confirm significant upregulation in the CPT-11-R cells in comparison to the expression in the LoVo cell line (Figure 4D). The *GNL3L* protein expression level was significantly increased in the CPT-11-R cells compared to that in the LoVo cell line (Figure 4E). 

Furthermore, we validated the miR-4454 expression patterns in the microsatellite stable CRC cell lines SW480 and SW620. The results showed that miR-4454 expression was significantly reduced in the metastatic SW620 cell line compared to the primary SW480 cell line (Figure 4F). Next, we identified the *GNL3L* mRNA and protein expression pattern in the SW480 and SW620 cell lines. The results showed that *GNL3L* expression was upregulated in the metastatic SW620 cell line compared to the primary SW480 cell line (Figure 4G,H). Together, the results showed that miR-4454 downregulation and *GNL3L* upregulation were inversely correlated in the chemoresistant CRC cell line.

### 2.5. GNL3L Is a Functional Target of miR-4454

**Figure 5:**
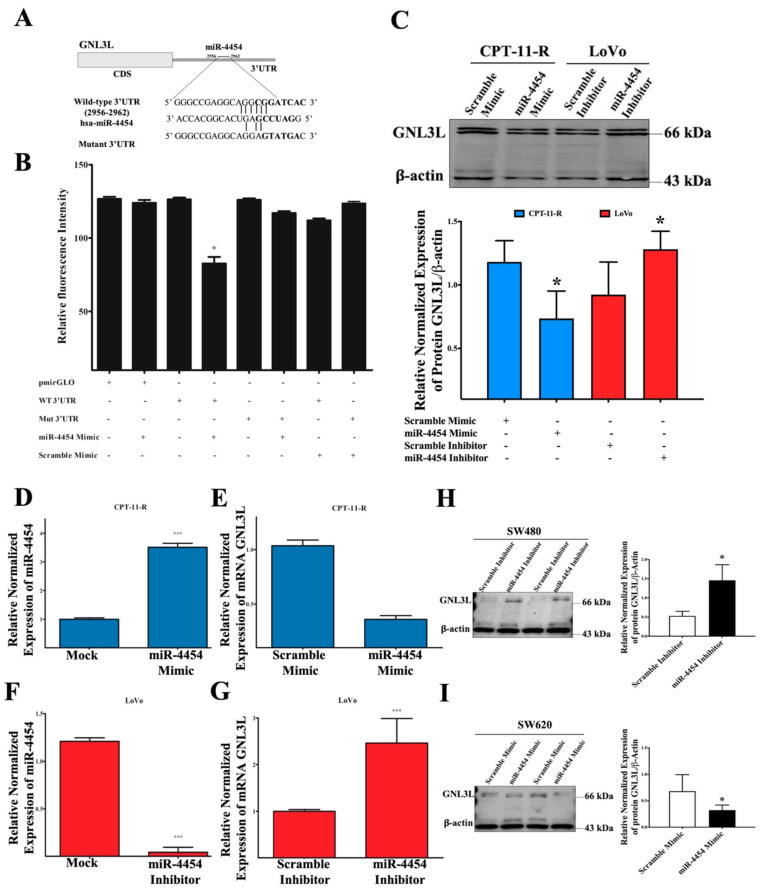
*GNL3L* is a functional target of miR-4454. (**A**) Schematic diagram of the miR-4454 binding target sequences of the wild-type and mutant 3’UTRs. (**B**) Luciferase reporter assay showing the relative luciferase intensity of wild-type and mutant *GNL3L* 3’UTR targeted by miR-4454 and the scramble. (**C**) Results from the Western blot analysis of *GNL3L* protein expression levels in CPT-11-R cells overexpressing miR-4454 and LoVo cells with inhibition of endogenous miR-4454. (**D**) Results from the q-RT-PCR analysis of CPT-11-R transient transfection efficiency of miR-4454 overexpression, with the same conditions for (**E**) *GNL3L* mRNA expression and (**F**) LoVo endogenous miR-4454 inhibition efficiency level as well as (**G**) for *GNL3L* mRNA expression level. (**H**,**I**) Results from the Western blot analysis of *GNL3L* protein expression level in SW480 (miR-4454 inhibitor) and SW620 (miR-4454 mimic) cells. The results are presented as mean ± standard error of the mean (*n* = 3). * *p* < 0.05, *** *p* < 0.001 compared to control.

To identify the effect of miR-4454 on the posttranscriptional suppression of the target gene, we performed luciferase reporter assay with a *GNL3L* wild-type and a 3’UTR mutant. The results showed that miR-4454 significantly reduced the luciferase signal unit in the group transfected with the wild-type miR-4454 target sequence (CGGATC) compared with the group transfected with the pmiRGLO empty vector control. Conversely, the miR-4454 effect was absent in the group transfected with the mutant miR-4454 target sequence (GTATGA) (Figure 5A,B). Furthermore, we analyzed the *GNL3L* protein expression level in the CPT-11-R cells. Interestingly, it was found that the transient overexpression of miR-4454 remarkably reduced the level of *GNL3L* protein compared with that of the scrambled transfection group, and conversely, the inhibition of endogenous miR-4454 in the LoVo cells increased the *GNL3L* levels (Figure 5C). Indeed, transient transfection of the miR-4454 mimic or inhibitor treatment group revealed that miR-4454 overexpression in the CPT-11-R cells significantly increased the miR-4454 expression levels and reduced the *GNL3L* mRNA levels (Figure 5D,E). LoVo cell inhibition of endogenous miR-4454 showed significantly reduced miR-4454 expression levels and increased *GNL3L* mRNA levels (Figure 5F,G). Furthermore, we used the microsatellite stable cell lines SW480 and SW620 to confirm that the inhibitor or mimic of miR-4454 significantly altered the *GNL3L* protein expression level (Figure 5H,I). 

### 2.6. MiR-4454 Suppresses Colon Cancer Cell Growth, Invasion, and Migration through the GNL3L and NF-κB Pathways

**Figure 6:**
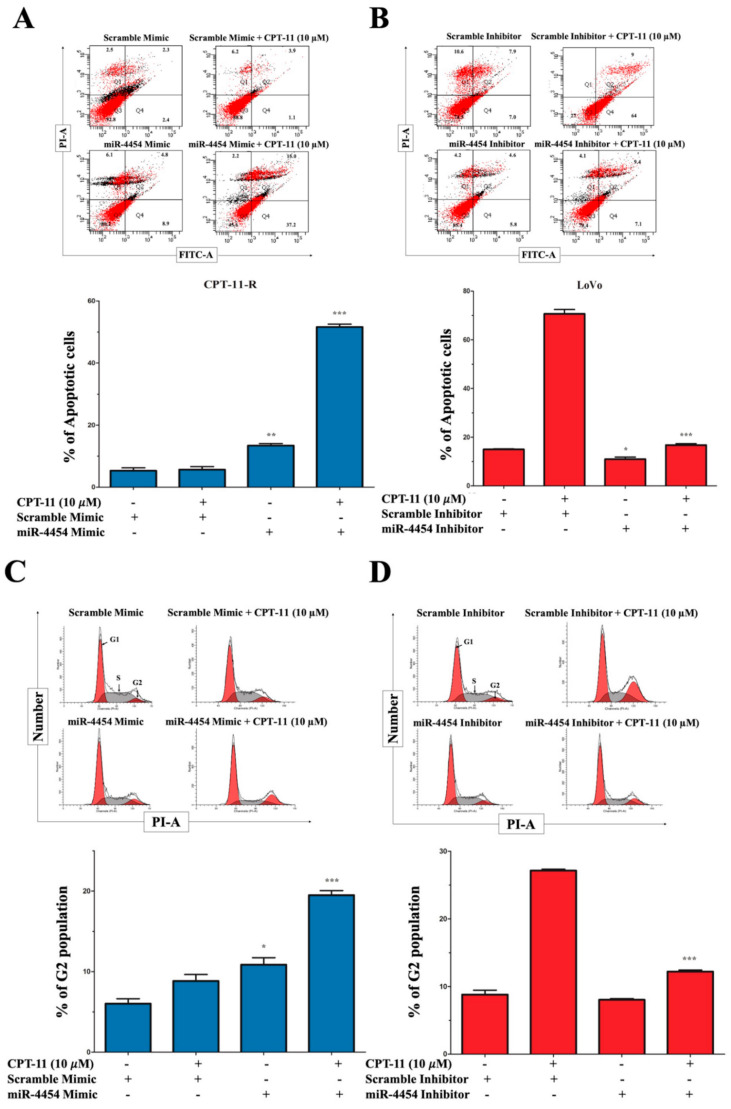

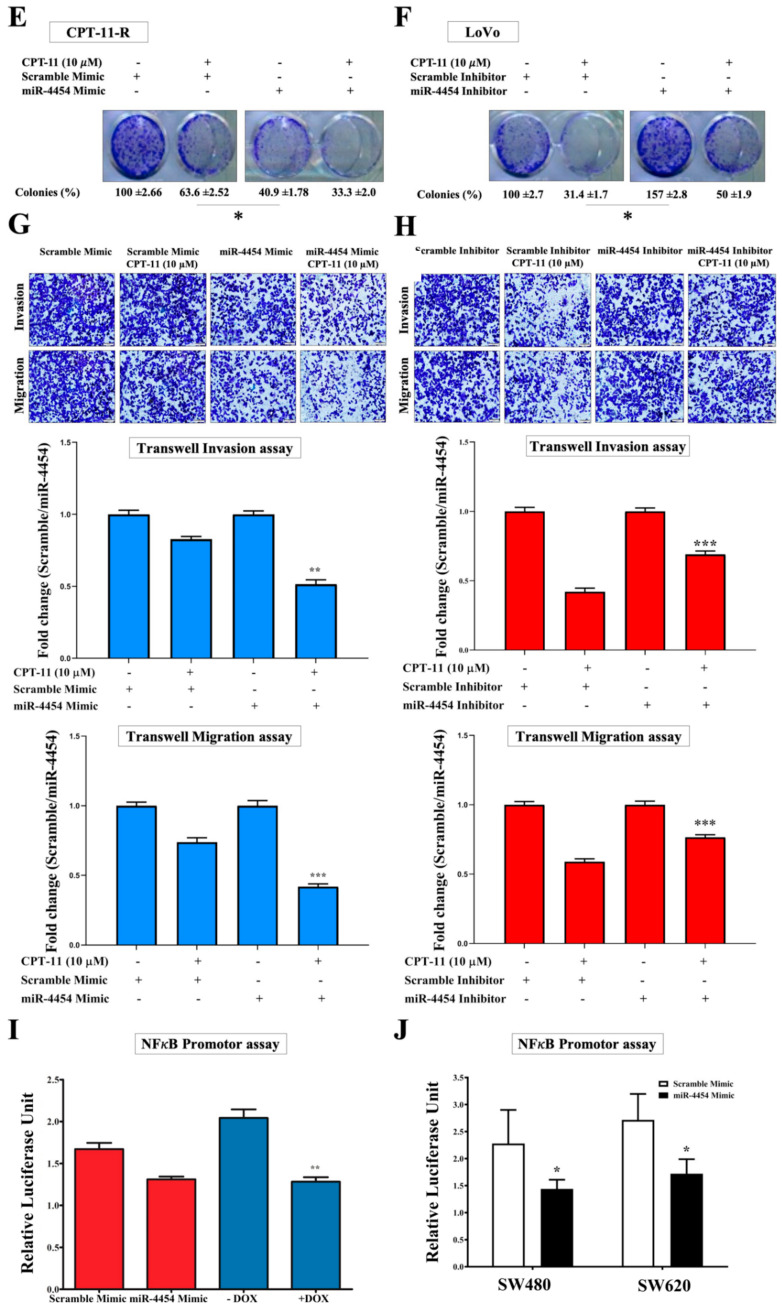
MiR-4454 suppresses colon cancer cell growth, invasion, and migration through the *GNL3L* and *NF-κB* pathways. (**A**) Annexin V apoptosis cell flow cytometry analysis for ectopic overexpression of miR-4454, which enhances the chemo sensitization effect of CPT-11-R cells treated with CPT-11 (10 µM) at 48 h time point (upper & lower). (**B**) Inhibition of endogenous miR-4454 promotes LoVo cell chemoresistance to CPT-11 (10 µM) at 48 h time point (upper & lower) and fold change normalized with scramble mimic for CPT-11-R and scramble inhibitor for LoVo cell line. (**C**) Cell cycle G2/M phase analysis during ectopic overexpression of miR-4454 in the CPT-11-R cells (upper & lower) and (**D**) during endogenous miR-4454 inhibition in LoVo cells (upper & lower). (**E**,**F**) Colony formation ability of CPT-11-R cells and LoVo cells. (**G**) Results from the invasion/migration analysis of ectopically overexpressed miR-4454 in CPT-11-R cells (upper & lower). (**H**) Results from the invasion/migration analysis of the inhibited endogenous miR-4454 in LoVo cells (upper & lower) (scale bar: 100 µm). (**I**,**J**) Results from the promoter assay analysis of the relative luciferase *NF-κB* promoter signal unit reduction in miR-4454-overexpressed groups. The results are presented as mean ± standard error of the mean (*n* = 3). * *p* < 0.05, ** *p* < 0.01, *** *p* < 0.001 compared to control.

We examined whether the ectopically overexpressed miR-4454 promotes the sensitivity of the CPT-11-R cells to the CPT-11 drug, and we found that miR-4454 inhibited cell invasion and migration and colony formation ability and increased the cell death rate in the presence of anticancer drugs. Overexpressed miR-4454 in the CPT-11-R cells enhanced chemosensitization, and when miR-4454-overexpressing cells were challenged with the CPT-11 drug (with EC_10_ concentration from the MTT assay of resistant cells), the apoptotic cell rate was increased compared to that in the controls. This finding indicated an increased chemosensitivity to CPT-11 drug treatment in the ectopic miR-4454-transfected CPT-11-R cells at 48 h (Figure 6A). Consistent with this observation, the inhibition of endogenous miR-4454 enhanced the chemoresistance of LoVo cells against the CPT-11 drug. Suppressed endogenous miR-4454 levels promote the chemoresistance of LoVo cells, showing that LoVo cells treated with the CPT-11 drug had an increased rate of apoptosis compared to that of the controls, and the inhibition of endogenous miR-4454 led to a lower rate of apoptosis compared to that of the controls. Cells with inhibited endogenous miR-4454 that were challenged with the CPT-11 drug (EC_50_ concentration was based on that of the LoVo cell MTT assays) showed a decreased rate of apoptosis compared to that of the controls. This finding implies an increased chemoresistance state for the LoVo cells transfected with the endogenous miR-4454 inhibitor and challenged with CPT-11 drug treatment for 48 h (Figure 6B).

To examine the critical role of *GNL3L* in the oncogenic-related factors of cell cycle progression, we overexpressed miR-4454 to promote G2/M phase arrest in the CPT-11-R cells and significantly promoted G2/M phase arrest with the CPT-11 drug (Figure 6C). Consistent with this observation, the inhibition of endogenous miR-4454 significantly protected the LoVo cells from the G2/M phase arrest induced by the CPT-11 drug (Figure 6D). Then, we studied the colony formation ability of the CPT-11-R cells overexpressing miR-4454 by challenging it with the CPT-11 drug and found that the CPT-11 drug significantly affected the colony formation of the CPT-11-R cells compared to that of the respective control group (Figure 6E). In the LoVo cells, the inhibition of endogenous miR-4454 significantly increased colony formation after CPT-11 drug treatment (Figure 6F). Moreover, using transwell invasion/migration assays, we demonstrated that miR-4454 overexpression in the CPT-11-R cells significantly reduced the invasion/migration ability after CPT-11 drug treatment (Figure 6G), and the inhibition of endogenous miR-4454 in the LoVo cells significantly increased the invasion/migration ability after CPT-11 drug treatment (Figure 6H). We further examined the effect of miR-4454 overexpression on *NF-κB* signaling in the LoVo, CPT-11-R miR-4454 stable, and SW480 and SW620 cell lines. We used the *NF-κB* luciferase promoter assay with miR-4454 overexpression in the LoVo, SW480, and SW620 cell lines, and the doxycycline-induced miR-4454 overexpression in the CPT-11-R stable cell line group showed that the *NF-κB* luciferase unit was significantly reduced (except for the slightly reduced LoVo cells) compared to that of the respective controls (Figure 6I,J).

### 2.7. Silencing GNL3L Attenuates Proliferation, Colony Formation, and Invasion/Migration

**Figure 7:**
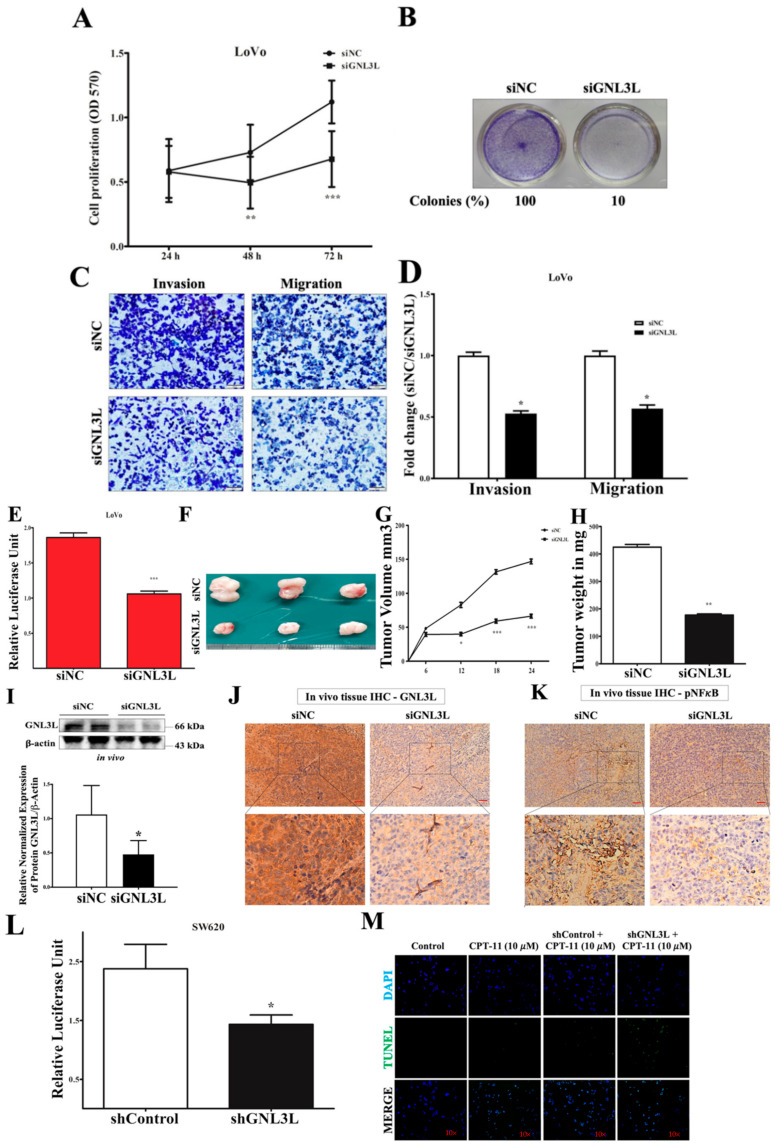

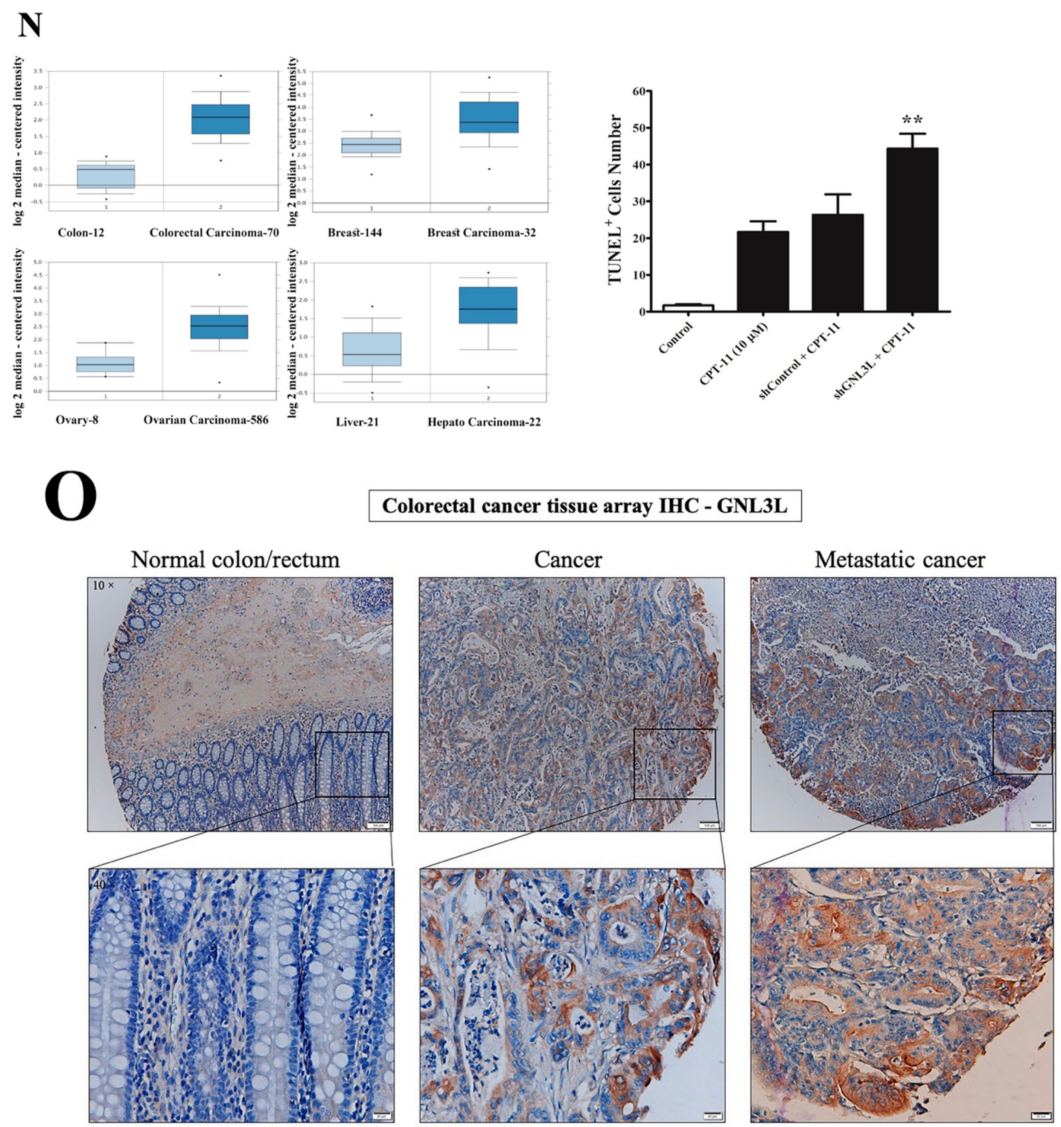
Silencing *GNL3L* imitates the effect of proliferation, colony formation, and invasion/migration. Results from (**A**) cell proliferation (OD 570) and Western blotting (**B**) colony formation. (**C**,**D**) Transwell invasion/migration assays (upper & lower) showing that silencing of *GNL3L* inhibited cell proliferation; the level of *GNL3L* silencing efficiency, colony formation, and invasion/migration compared to the control is presented (scale bar: 100 µm). (**E**) Relative luciferase *NF-κB* promoter signal unit reduction in *GNL3L*-silenced LoVo cells. (**F**,**G**,**H**) Tumor volume measurements. (**I**,**J**) Western blot and IHC analysis of *GNL3L* protein expression level, (**K**) IHC analysis of *NF-κB* expression (scale bar: 50 µm). shRNA-mediated silencing *GNL3L* in metastatic SW620 cell line. Results from (**L**) *NF-κB* promoter assay, and (**M**) TUNEL assay (10×). (**N**) Results from the Oncomine database analysis of *GNL3L* expression levels. (**O**) Representative image showing an inverse correlation of *GNL3L* expression level between adjacent tissue and cancer or metastatic tissue as determined by IHC analysis using a commercial CRC array (US Biomax, Inc.) (scale bar: 20 µm). (The results are presented as mean ± standard error of the mean (*n* = 3). * *p* <0.05, ** *p* <0.01, *** *p* <0.001 compared to control.

To investigate the important role of *GNL3L* in colon cancer cells, we employed the Ambion^®^ predesigned siRNA to silence endogenous *GNL3L* in the LoVo colon cancer cells. Cell proliferation assays showed that the depletion of *GNL3L* remarkably suppressed LoVo colon cancer proliferation, by ~40% at different time points (Figure 7A). Moreover, colony formation was significantly affected in the *GNL3L*-silenced group compared to that in the negative control group (Figure 7B). The Western blot analysis revealed that >60% of the endogenous *GNL3L* was reduced in the LoVo cells (Figure 7C). Moreover, we used a transwell assay for invasion/migration capacity analysis, and it showed that the depletion of endogenous *GNL3L* significantly affected the invasion/migration ability of the metastatic LoVo cell line (Figure 7C,D). The findings of previous studies on the relationship between *GNL3L* and *NF-κB* promoter assays and that of our studies on colon cancer were consistent in that the depletion of *GNL3L* remarkably reduced the luciferase signals. These data support the notion that the depletion of *GNL3L* is attributable to *NF-κB* promoter activation (Figure 7E). 

We further examined the functional effect of *GNL3L* in colon cancer cells in vivo by subcutaneously injecting LoVo cells pretreated with a predesigned *GNL3L* silencer that is activated in vivo, and the systemic delivery of predesigned siRNA was performed thrice with one-week intervals. The results showed that the silenced *GNL3L* reduced the tumor volume significantly in the LoVo cell group compared with that of the control group (Figure 7F–H), and we further confirmed *GNL3L* expression using Western blotting and immunohistochemistry (Figure 7I,J) and *NF-κB* expression using IHC (Figure 7K). Furthermore, we confirmed the *GNL3L* silencing in the microsatellite stable metastatic SW620 colon cancer cell line using shRNA. The results from the shRNA silencing in *NF-κB* promoter assays showed a significant reduction in luciferase unit compared to that of the controls (Figure 7L). The Terminal deoxynucleotidyl transferase dUTP nick end labeling (TUNEL) assay results showed a significant increment of TUNEL-positive cells in sh*GNL3L* treated with CPT-11 (10 µM) at 24 h (Figure 7M). Based on the Oncomine database (https://www.oncomine.org/), a higher *GNL3L* expression was found in tumors belonging to four different cancer data sets (colorectal, liver, breast, ovarian) (Figure 7N), in comparison to that of normal tumors. Given that there is an inverse relationship between the normal and cancer tissue expression of *GNL3L*, it is interesting to evaluate their expression patterns in clinical samples. We performed IHC experiments to evaluate *GNL3L* expression using a commercial human CRC (with respective metastatic tumor and adjacent normal cells) tissue array (CO992a). The results demonstrated that the low expression of *GNL3L* in adjacent normal patient tissue was significantly and inversely correlated with the high expression of *GNL3L* in cancer and metastatic cancer patient tissues (Figure 7O and Table 1). These findings confirmed that *GNL3L* has a tumorigenic effect on colon cancer cells.

### 2.8. Doxycycline-Induced Expression of miR-4454 Suppresses Colon Cancer Growth 

To extend the study and determine the precise functional role of miR-4454 in tumor growth, the apoptotic rate, and the invasion/migration ability of the CPT-11-resistant colon cancer cells, we performed doxycycline (Dox) induction of miR-4454. RFP expression levels and the qRT-PCR results revealed that the expression of miR-4454 was increased at different concentrations of Dox at 72 h (Figure S1A,B). Consistent with this observation, the Western blot results showed that the miR-4454-induced group had significantly reduced *GNL3L* protein expression compared to the untreated group (Figure S1C). The colony formation and cell proliferation assay results showed that the Dox-induced miR-4454 group had significantly reduced colony formation ability and lower cell proliferation rate compared to the untreated group and the scramble control groups with or without Dox treatment (Figure S1D,E). Consistent with these results, the invasion/migration ability was also affected in the same Dox-induced groups (Figure S1G). Moreover, we analyzed the first-line anticancer therapy with different apoptotic induction rates in the groups with or without Dox treatment. The results demonstrated that the miR-4454-induced group had significantly increased apoptosis rates when treated with 5-FU, CPT-11, or OXA (Figure S1F).

**Figure 8:**
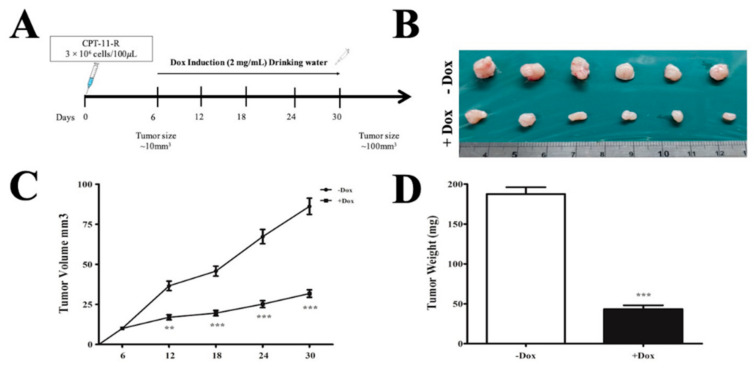

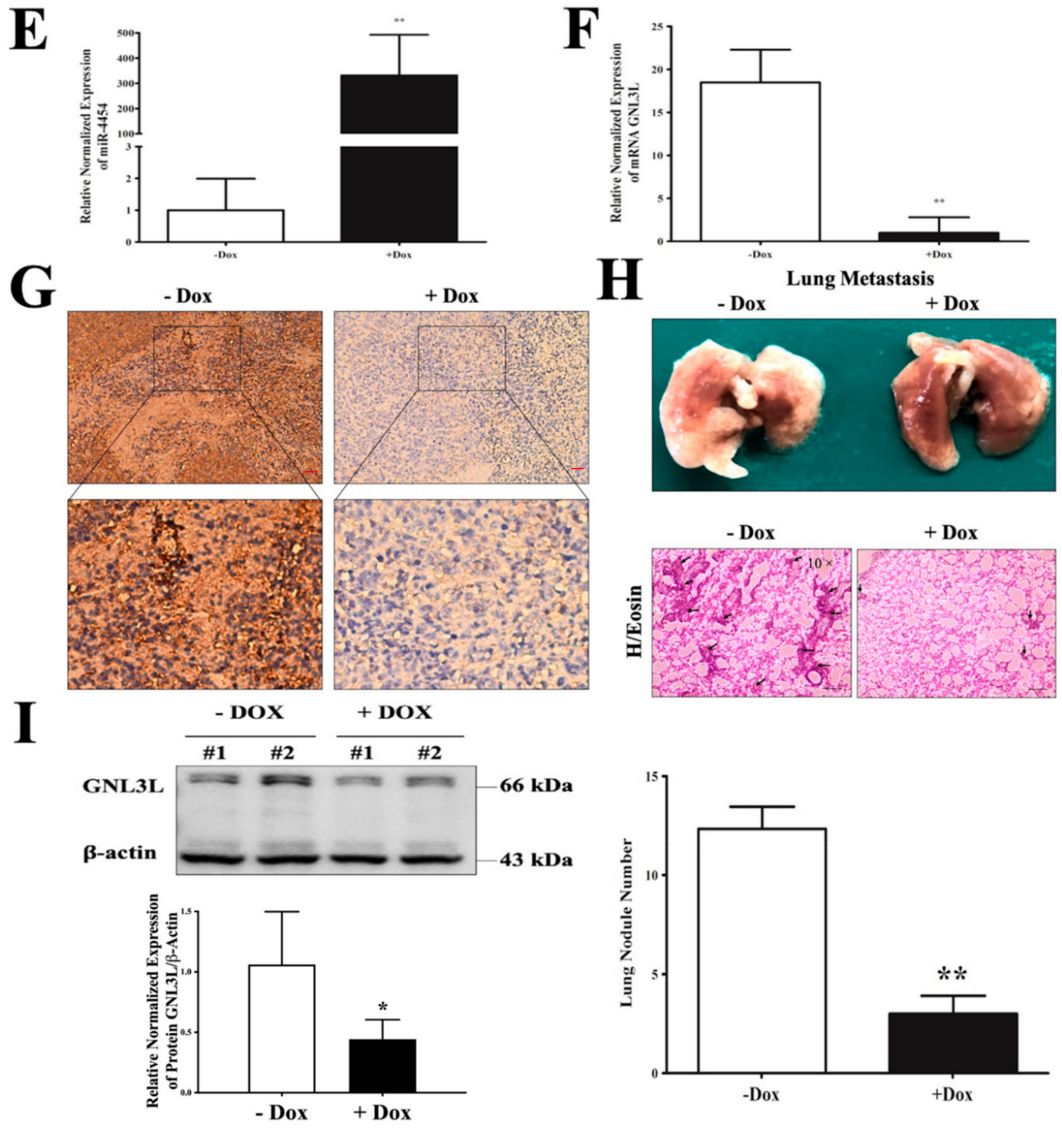
Doxycycline-induced expression of miR-4454 suppresses colon cancer growth. (**A**) Schematic overview of the doxycycline-inducible miR-4454 expression in mice and the tumor suppression effect of the resistant colon cancer cells injected subcutaneously in nude mice. (**B**,**C**) Tumor volume measurements (mm^3^). (**D**) Tumor weight measurement (mg). (**E**) −Dox or +Dox tumor miR-4454 expression level and (**F**) *GNL3L* expression level. (**G**) IHC analysis of *GNL3L* protein expression level (scale bar: 50 µm). (**H**) Lung metastasis node formation morphology and hematoxylin and eosin stain (the arrow represents the node tumors formed). (**I**) Western blot analysis of GNL3L expression level. The results are presented as mean ± standard error of the mean (*n* = 3). * *p* < 0.05, ** *p* < 0.01, *** *p* < 0.001 compared to control.

Furthermore, we examined the consistency of the functional role of the miR-4454-inducible CPT-11-resistant cells in a nude mouse model (Figure 8A). After six days of experiments, the Dox-induced tumor size was measured in the groups inducted and not inducted with Dox. The results showed that the Dox-inducted group had tumors of significantly reduced size compared to those in the group not inducted with Dox (Figure 8B,C). After the tumors were excised, the final tumor weight was significantly reduced in the Dox-inducted group compared to that of the group not inducted with Dox (Figure 8D). We further validated miR-4454 and *GNL3L* expression levels in the excised tumor tissue from each group. The results showed that the Dox-inducted group had a significantly increased miR-4454 expression level and a reduced *GNL3L* expression level (Figure 8E,F). Furthermore, we confirmed the *GNL3L* expression level using IHC analysis (Figure 8G) and Western blot analysis (Figure 8I). We also collected the lungs from each group and examined for metastatic node formation using hematoxylin and eosin stain. The results showed that the group not inducted with Dox had significant metastasis compared to the Dox-inducted group (Figure 8H).

### 2.9. Schematic Representation of the miR-4454/GNL3L/NFκB Pathway

**Figure 9:**
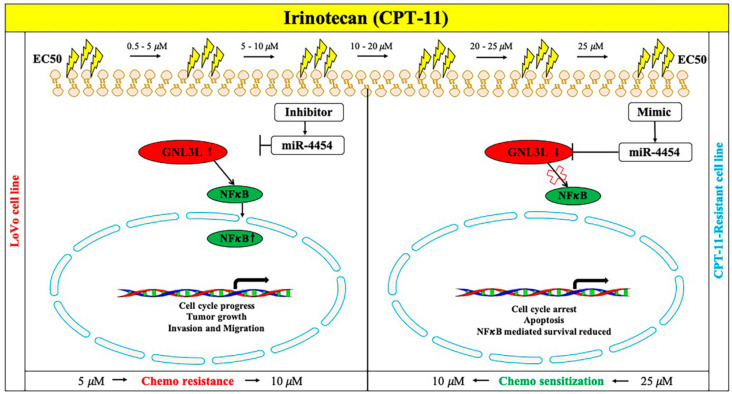
Schematic representation of the miR-4454/*GNL3L*/*NFκB* pathway.

The schematic diagram of the present study (Figure 9) shows that chemoresistance-associated silencing of miR-4454 promotes CRC aggression, reverted through the overexpression of miR-4454 by targeting *GNL3L* (tumor initiation or maintenance-related gene) and the *NFκB* pathway.

## 3. Discussion

Adjuvant chemotherapy consisting of 5-FU, CPT-11, and oxaliplatin currently remains the first-line chemotherapy treatment for patients with high-grade CRC. However, the chemoresistance leading to treatment failure and relapse is still a critical problem for identifying the subpopulation of patients who are most likely to respond to targeted therapy. If the mechanism that mediates variations in patient response to chemotherapy could be predicted, then effective treatment strategies for non-responders could be formulated. Evidence shows that the modulation of microRNA can alter the response to chemotherapy and reverse microRNA-mediated responses, potentially enhancing the efficacy of antitumor therapy [6,20,21,22].

Chemotherapy response is closely correlated to the functional status of the microRNA expression level or that of the microRNA-regulated gene. Notably, we found that miR-4454 was downregulated in chemoresistant CRC and that one of the target genes, *GNL3L*, had a higher expression level in the CPT-11-R cell line compared to that in the LoVo colon cancer parental cell line. Downregulation of miR-4454 mediates chemoresistance and cell survival, demonstrating that the miR-4454 target gene, *GNL3L*, may play an important role in CRC chemoresistance and survival. The miR-4454 was previously reported in the LIM1863 colon carcinoma cell line to be secreted through EVs and exosomes [20].

*NF-κB*-mediated resistant clone survival has been highlighted as a key regulatory process against CPT-11 [10,23,24,25]. *GNL3L* promotes tumorigenesis, cell cycle regulation, and antiapoptosis through *NF-κB* activation. There has been evidence that microRNAs could modulate drug-resistant genes, thereby playing important roles in chemosensitivity [6,12,13,14,19,21,26,27,28], and the *GNL3L* gene can be affected by *LDOC1*, but this gene is mostly hypermethylated in cancers [18]. The overexpression of miR-4454 can increase sensitivity to CPT-11 in colon cancer. These findings suggest that the dysregulation of miR-4454 may be associated with drug resistance. We have shown that highly expressed *GNL3L* was significantly associated with CRC progression and metastasis. We found that, when CPT-11 is the first-line therapy, CPT-11-R cells are protected from relatively high rates of arrest in the G2/M phase compared to the LoVo parental colon cancer cell line [17].

Nonetheless, it makes sense that the tumor-suppressor miR-4454 is involved in attenuation of *GNL3L*. Indeed, we found *GNL3L* overexpression in chemoresistant CRC cells. *GNL3L* overexpression was positively correlated with the progression of cancer and metastasis as determined using protein expression measures in human CRC tissue array samples. These findings demonstrate that miR-4454 downregulation can, at least partially, account for *GNL3L* overexpression in colon cancer and provide important insights into the mechanisms underlying *GNL3L* overexpression in chemoresistant CRC. However, there has been no study regarding the microRNA targeting *GNL3L*.

Our study revealed that overexpression of miR-4454 promoted G2/M phase arrest and proapoptosis in CPT-11-R cells when CPT-11 was the first-line therapy. We also observed that the inhibition of miR-4454 in the LoVo cells inversely correlated with miR-4454-overexpressing CPT-11-R cells. Interestingly, our in vivo study showed that a Dox-inducible system designed to overexpress miR-4454 in the CPT-11-R cells led to decreased tumor volume compared to that found in groups not overexpressing miR-4454, resulting in increased sensitivity to chemotherapy drugs. Thus, previous findings [10,17,18,21] and those from the current study indicate that the *GNL3L* expression level might be a predictive factor of chemoresistance in CRC patients. In summary, miR-4454 overexpression in chemoresistant CRC raises the possibility that the inhibition of *GNL3L* is an efficient therapeutic approach in overcoming chemoresistance.

## 4. Materials and Methods

### 4.1. Cell Culture

CRC cell lines LoVo, SW480, and SW620 were purchased from Bioresource Collection and Research Center (Hsinchu, Taiwan). The CPT-11-resistant clones developed from the LoVo cell line were cultured in RPMI 1640 (Gibco^®^ Grand Island, NY, USA) supplemented with 10% fetal calf serum (FCS; HyClone, Logan, Australia). All cells were incubated in a humidified chamber with 5% CO_2_ at 37 °C. The cell medium was changed 48 h after subculture. Dulbecco’s phosphate-buffered saline (PBS; Gibco^®^, Auckland, New Zealand) was used to wash the culture plate.

### 4.2. 3-[4,5-Dimethylthiazol-2-yl]-2,5-diphenyltetrazolium Bromide Assay

LoVo or CPT-11-R colon cancer cells seeded at a density of 1 × 10^5^ cells/well were grown in 24-well plates containing RPMI 1640 (10% FBS). Twelve hours later, the cells were treated with different concentrations (5, 10, 15, 20, and 25 µM) of the CPT-11 drug (AdooQ Bioscience LLC, Irvine, CA, USA) following LoVo (48 h) or CPT-11-R (72 h) time point. For the cell proliferation assay, after treatment with siRNA, Dox with or without cells was maintained, and at different time intervals, MTT was performed. Basically, for MTT assay, MTT (Sigma-Aldrich, St. Louis, MO, USA) concentration (5 mg/mL stock solution in PBS, diluted with culture medium to a final concentration of 0.5 mg/mL) was added. After 4 h of incubation at 37 °C, the media were removed, and the formazan crystals were solubilized with dimethyl sulfoxide (DMSO) (Sigma-Aldrich). The absorbance was measured at 570 nm (using an automated microplate reader) to determine the intensity of the formazan crystal, which is proportional to cell survival.

### 4.3. Flow Cytometry for the Annexin V Apoptosis Assay

For the flow cytometry Annexin V apoptosis assay, the LoVo cells and CPT-11-R clones were treated with different concentrations (5, 10, 15, 20, and 25 µM) of the CPT-11 drug for 48 h (LoVo cells) and 72 h (CPT-11-R clones). The cells were collected and stained using an Annexin V-FITC apoptosis detection kit (BD, Biosciences, San Jose, CA, USA) according to the manufacturer’s protocol. Flow cytometry was performed at the Fluorescence Activated Cell Sorting (FACS) Core Facility, China Medical University, Taiwan, using a FACS Canto^TM^ system (BD, Biosciences). Cells were gated to obtain cell singlets after cells in each quadrant of the fluorescein isothiocyanate (FITC-A) versus PI plot. For convenience, only 10,000 cells of the 50,000 cells visualized were counted. The cell death rate was calculated as 100%(1–(Q3_Drug_/Q3_Control_)).

### 4.4. Cell Cycle Analysis

For the cell cycle analysis, LoVo cells and CPT-11-R clones were treated with different concentrations (5, 10, 15, 20, and 25 µM) of the CPT-11 drug for 48 h (LoVo cells) and 72 h (CPT-11-R clones). After treatment, the cells were harvested and then washed with cold PBS. The cells were further fixed with 70% ice-cold ethanol at 4 °C overnight. After incubation in PBS containing 10 mg/mL PI and 0.5 mg/mL RNase A for 15 min at room temperature (RT), the fixed cells were washed with ice-cold PBS thrice. FACS Canto^TM^ flow cytometry was used to determine the DNA content of the labeled cells. For all cycle events, *n* = 10,000 cells.

### 4.5. Microarray Profiling and Target Prediction

Expression profiling of the LoVo cells versus the CPT-11-R clones was performed using total RNA (all with RIN = 10) on the microRNA array by the Phalanx Biotech Co., Ltd. (Taipei, Taiwan). A human microRNA OneArray^®^ platform HmiOA v7 (Taipei, Taiwan), which provides 100% miRbase v21 coverage (www.mirbase.org) by a one-color approach, was employed for universal microRNA coverage. To identify putative miR-4454, target sequences were obtained from the TargetScan 7.1 online software (www.targetscan.org) for all annotated human transcripts. Furthermore, we used the Oncomine database of gene expression analysis for the four cancer data sets analyzed (colorectal, breast, liver, ovarian) that were compared against the expression levels of normal cells (https://www.oncomine.org/) [29].

### 4.6. Western Blot Analysis

Western blot analysis and protein extraction were performed according to standard procedures [30]. The cells were lysed in lysis buffer (50 mM Tris-base (pH 7.5), 0.5 M NaCl, 1 mM EDTA (pH 8), 1 mM β-mercaptoethanol, 1% NP40, 1% glycerol, and protease inhibitor (Roach Molecular Biochemical, Mannheim, Germany)). The protein concentrations were determined using the Bio-Rad Protein Assay Kit following the Bradford method (Bio-Rad, Munich, Germany). The samples were separated by 10%–15% sodium dodecyl sulfate-polyacrylamide gel electrophoresis (SDS-PAGE) and transferred onto polyvinylidene difluoride (PVDF) membranes (Millipore, Billerica, MA, USA). The membranes were blocked with 5% skim milk (5% milk in 1× TBST (0.13% Tris-base, 1.47% Nacl, and 0.05% Tween 20) for 1 h at RT and incubated with specific primary antibodies (listed in Table S3) at 4 °C overnight. Thereafter, the membranes were washed with TBST and incubated with a secondary antibody for 1 h at RT, and the signals were measured with Western chemiluminescent HRP substrate (Millipore). The Western blot analysis was performed using an iBright 1500 luminescent image analyzer (ThermoFisher scientific, St. Louis, NY, USA).

### 4.7. RNA Extraction, microRNA First-Strand Synthesis, and qRT-PCR

Total RNA from the cells was extracted and purified using an RNA MiniPrep kit (Zymo Research Corporation, Irvine, CA, USA) according to the manufacturer’s guidelines. The RNA quality was ensured by NanoSample (260/280 = 1.8). The microRNA expression profile was analyzed with RT-PCR (CFX96^TM^ Quantitative Real-Time system, Bio-Rad) according to the protocol from the manufacturer of the Mir-X^TM^ microRNA first-strand synthesis (cDNA) kit (TaKaRa, Ohtsu, Japan). MicroRNA forward primers (PROTECH, Taipei, Taiwan) and reverse primer strands were provided in a Mir-X^TM^ microRNA first-strand synthesis (cDNA) kit, and SYBR Green PCR Master Mix (Bio-Rad) was used according to the manufacturer’s protocol. For mRNA detection, the cDNA was synthesized using an iScript^TM^ cDNA synthesis kit (Bio-Rad), and qRT-PCR was performed using the SYBR Green PCR Master Mix (Bio-Rad). We used a CFX96^TM^ Quantitative Real-Time system following the manufacturer’s guidelines with a total reaction volume of 20 µL containing 10 µL SYBR Green PCR Master Mix, 300 nM (each) forward and reverse primers, and 100 ng cDNA. The reactions were incubated in a Bio-Rad qRT-PCR 96-well tube or 8-well tube at 95°C for 3 min, followed by 40 cycles of 95 °C for 15 sec, 55 °C for 30 sec, and 72 °C for 30 sec. All reactions were run in triplicate. The cycle number at which the reaction crossed the threshold cycle (C_t_) was determined for each gene. The relative amount of each microRNA to U6 rRNA was measured using the 2^ΔCt,^ equation, in which ΔC_t_ = (C_t_ microRNA – C_t_ U6 rRNA).

### 4.8. Extracellular Vehicle RNA Isolation

The EVs were isolated from in LoVo and CPT-11-R cell culture media according to the instructions of the Exo-spin™ exosome purification kit (Cell Guidance Systems). LoVo and CPT-11-R cells were cultured (with exosome-free serum medium) in 100 mm plates. After the cells reached 70% confluence, the culture medium was collected in a 50 mL falcon tube and centrifuged at 300× *g* for 10 min to remove the cells, and the supernatant was transferred to a new tube and centrifuged at 16,000× *g* for 30 min to remove any remaining cell debris. The supernatant was transferred to a new tube, and 20 mL of supernatant was added to 10 mL of Exo-spin™ buffer and was mixed well by tube inversion, and then, the mixture was incubated at 4 °C overnight. Next, the mixture was centrifuged at 16,000× *g* for 1 h, and the supernatant was carefully aspirated and then discarded. The EV-containing pellet was resuspended in 500 µL of TRIzol LS reagent (Thermo Fisher Scientific), and 200 µL of chloroform was added and mixed by inverting the tube before it was incubated for 10 min. Phase separation was performed by centrifugation at 12,000× *g* at 4 °C for 15 min. The upper aqueous phase was collected and mixed with RLT buffer (3.5 × volume), absolute ethanol (2.5 × volume), and sodium acetate (3 M, pH 5.5) (0.1 × volume), and the sample was mixed and incubated at –80 °C overnight and then centrifuged at 16,000× *g* at 4 °C for 5 min. The pelleted EV RNA was purified using a Direct-zol RNA MiniPrep kit (Zymo Research Corporation, Irvine, CA, USA) according to the manufacturer’s guidelines.

### 4.9. MicroRNA and siRNA or shRNA Transfection Experiments

For microRNA and siRNA or shRNA experiments, cells were seeded in a well plate or culture petri dish depending on the experiments. MicroRNA mimic or inhibitor and scramble mimic or inhibitor were purchased from RiboBio (Guangzhou, China), siRNA *GNL3L* and control were purchased from Life Technologies (Ambion® predesigned siRNA, Carlsbad, CA, USA), shRNA *GNL3L* and shRNA control were purchased from the National RNAi Core Facility (Academia Sinica, Taipei, Taiwan), and plasmid transfection was performed using JetPRIME^®^ reagents (Polyplus-transfection, Brant, France) according to the manufacturer’s guidelines.

### 4.10. miR-4454 Dox-Inducible Expression Vector and Gene Reporter Plasmid Construction

The Dox-inducible miR-30 pSBInducer vector, scramble pSBinducer vector, and pCMV-SB100XCO helper plasmid were provided by Prof. Claus Lindbjerg Andersen (Department of Molecular Medicine, Aarhus University Hospital, Aarhus, Denmark). The mature human miR-4454 sequence and the universal miR 30 sequence were combined and used as a template to produce inserts for PCR using universal miR 30 primers (Table S2). From this process, the pSBinducer was inserted into the mature sequence of miR-4454 using the XhoI and EcoRI restriction sites. For the gene reporter assay, a construct containing the 3’UTR of the *GNL3L* plasmids (wild type and mutant) was made using the annealing method to produce an insert (Table S1) containing the restriction digested product, which was inserted into the pmiRGLO vector using the same restriction XhoI and SalI sites and restriction enzymes, that was confirmed by colony PCR and restriction digestion. At all steps, the plasmid DNA was purified with Qiagen midi plasmid purification kit (Qiagen, Hilden, Germany) and confirmed by sequencing and restriction digestion to ensure that the correct insert was formed.

### 4.11. Luciferase Reporter Assay

Luciferase constructs were created for 3’UTR sequences (Table S1) of the *GNL3L* wild type and the mutant using a pmirGLO empty vector (Promega, Madison, WI, USA) and a promoter luciferase *NF-κB* construct (pGL4.32 (luc2P/*NF-κB*-RE/Hygro)) (Promega, Madison, WI, USA) for the induction with 20 ng/µL of TNFα for 5 h. The LoVo cells were seeded into a 12-well plate and co-transfected with the pmiRGLO-3’UTR or *NF-κB* promoter plasmid, the miR-4454 mimic, si*GNL3L*, or the respective control using JetPRIME^®^ reagents according to the manufacturer’s guidelines. Luciferase activities were detected using the dual-luciferase assay kit (Promega, Madison, WI, USA).

### 4.12. Generation of miR-4454 pSBinducer Cells

To generate miR-4454 pSBinducer cells, the Mads Heilskov Rasmussen [31] protocol was followed, and the miR-4454 Dox-inducible stable cell line was produced. The cells were transfected with 1500 ng pSBinducer (scramble or miR-4454) and 1500 ng pCMV-SB100XCO helper plasmid (or the negative control, pUC19 DNA) using 15 µL of JetPRIME^®^ reagent according to the manufacturer’s guidelines. After 24 h, the transfected cells were treated with a puromycin concentration of 2 µL mL^−1^ for 5 days to eliminate the control transfected cells. We used the RFP fluorescence marker to select the cell populations expressing miR-4454 or the scramble after Dox induction. These cells were frozen and used for subsequent experiments with a low passage cell population.

### 4.13. Transwell Invasion/Migration Assay

The in vitro cell invasion (chamber coated with Matrigel 200 µL) and migration assay was performed [32] using transwell chambers (8 μm pore size; SPLInsert™ hanging 24-well plate). A total of 5 × 10^4^ cells for the invasion assessment and 2 × 10^4^ cells for the migration assessment were plated in the upper chamber with serum-free medium. The medium containing 10% FBS in the lower chamber served as a chemoattractant. After 48 h, non-invading and nonmigrating cells were removed using cotton swabs from the upper face of the filters, and the migratory cells located on the lower side of the chamber were stained with crystal violet, air-dried, photographed, and counted. Images of four random fields at 20× magnification were captured from each membrane, and the number of invaded/migratory cells per field was counted.

### 4.14. Colony Formation Assay and Hematoxylin and Eosin Stain

Colony formation assays was performed with LoVo and CPT-11-R cells. Basically, 0.25% trypsin was used to harvest cells, and thereafter, a single cell suspension was dispersed in 10% FBS. The cells were seeded into 6- or 24-well plates at a density of 500 cells/well and cultured in a 5% CO_2_ atmosphere at 37 °C for 2–4 weeks. When cell clones appeared, they were fixed with 4% paraformaldehyde for 10–45 min, stained with Giemsa or 0.5% crystal violet for 10–40 min and counted under a light microscope. For lung metastasis node formation analysis, fixed tissue was stained with hematoxylin (Vector Laboratory, Burlingame, CA, USA) and eosin (Sigma-Aldrich).

### 4.15. Immunohistochemistry 

The commercial CRC tissue array (CO992a) was purchased from US Biomax (US Biomax, Inc., Rockville, MD, USA). The slides were incubated with the primary polyclonal anti-*GNL3L* antibody (ab94862, Abcam, Cambridge, MA, USA) at a 1:50 dilution ratio. Immunohistochemistry (IHC) was performed according to the manufacturer’s protocol.

### 4.16. In Situ TUNEL Assay

TUNEL staining for cells was based on fluorescein-dUTP for the labeling of DNA strand breaks by fluorescence microscopy described by the manufacturer’s protocol (Roche Molecular Biochemical, Mannheim, Germany). The number of apoptotic cells was counted by TUNEL^+^ cells.

### 4.17. Dox Induction In Vivo Tumor Studies 

Five-week-old BALB/C-NU/NU male mice purchased from BioLASCO, Taiwan, were used for the subcutaneous injection model. The experimental protocol (CMUIACUC-2019-237) was approved by the Institutional Animal Care and Committee of China Medical University. The LoVo cells (3 × 10^6^) were injected into nude mice (N = 3 per group). For gene silencing, the *GNL3L* group was administered with si*GNL3L* (50 µM in 25 g mice) according to the manufacturer’s protocol (Ambion®, Life Technologies, Carlsbad, CA, USA). The Dox-inducible CPT-11-R cells expressing miR-4454 (3 × 10^6^) were injected subcutaneously into the nude mice. After 6 days, the nude mice were separated into two groups (N = 6 per group). To induce miR-4454 expression, 2 mg/mL doxycycline was added to the drinking water, and the water was changed every 2 days for a 3-week period [33,34]. The other group of mice was not induced with Dox. The tumor size was measured using a caliper in 6-day intervals. The animals were housed and experimentally handled in a pathogen-free environment, and 12/12 day-night cycle was maintained according to the committee. The tumor volume was calculated using the formula volume = length × width 2/2. All animals were sacrificed 30 days after the cell injection. The tumors were further analyzed by Western blotting and by miR-4454 and *GNL3L* gene expression.

### 4.18. Statistical Analysis

The data are expressed as mean ± standard error of the mean. The results were compared using the unpaired Student t-test in Excel 2016 (Microsoft Corp., Redmond, WA, USA) and/or Prism GraphPad 5 wherever applicable. *P* values lower than *p* < 0.05, *p* < 0.01, and *p* < 0.001 were considered statistically significant.

## 5. Conclusions

In this study, we demonstrated that the loss of miR-4454 expression in a resistant clone prominently promoted tumor initiation, metastatic outgrowth, and resistant status through the *GNL3L/NFκB* signaling axis against anticancer drug therapy. Further, miR-4454 may be explored as a promising therapy for anticancer-resistant clones.

## Figures and Tables

**Table 1:** The expression of GNL3L in CRC compared to normal colon tissues.

Group	Cases (n)	*GNL3L* Expression		𝝌2	* *p* Value
		Low	High		
		Expression	Expression		
Normal colon/rectum	33	25 (76%)	8 (24%)		
Cancer	33	11 (33%)	22 (67%)	11.98	<0.0003
Metastatic cancer	33	5 (15%)	28 (85%)	24.44	<0.0001

CRC, colorectal cancer; metastatic cancer (lymph node); normal colon tissue 1.5 cm away from the tumor. *χ^2^ test was applied to assess the expression of GNL3L in CRC and normal colon tissues.

## Supplementary Materials

The following are available online at https://www.mdpi.com/2072-6694/12/5/1231/s1, Figure S1: Figure depicting single clone selection, Table S1: List of primers for qPCR; Table S2: List of primers for cloning; Table S3: Antibody list.
