# Supplementary material for: Chemoresistance-Associated Silencing of miR-4454 Promotes Colorectal Cancer Aggression through the GNL3L and NF-κB Pathway
Source: Cancers (Basel). 2020 May 14;12(5):1231. doi: 10.3390/cancers12051231 (PMC7281507; doi:10.3390/cancers12051231)
Supplement: Supplementary file 1 [file cancers-12-01231-s001.tgz › cancers-763346 supplementary final 2/cancers-763346 supplementary figure table layout.pdf]

cancers

# Supplementary Materials:

**Thetchinamoorthy Kannathasan, Wei-Wen Kuo, Ming-Cheng Chen, Vijaya Padma Viswanadha, Chia-Yao Shen, Chuan-Chou Tu, Yu-Lan Yeh, Mahalakshmi Bharath, Marthandam Asokan Shibu and Chih-Yang Huang**

**Table 1.** List of primers for qPCR.

| No. | ID Name | Sequences |
|---|---|---|
| 1. | hsa-miR-30c-1-3p | CTGGGAGAGGGTTGTTTACTCC |
| 2. | hsa-miR-4792 | CGGTGAGCGCTCGCTGGC |
| 3. | hsa-miR-6511b-5p | CTGCAGGCAGAAGTGGGGCTGACA |
| 4. | hsa-miR-4454 | GGATCCGAGTCACGGCACCA |
| 5. | hsa-miR-1914-3p | GGAGGGGTCCCGCACTGGGAGG |
| 6. | hsa-miR-5191 | AGGATAGGAAGAATGAAGTGCT |
| 7. | Forward – GNL3L | ACTCTGCCCACCCATCTCA |
| | Reverse – GNL3L | TTCAAGTGGGTCCGTGTCA |
| 8. | Forward - Xho1- WT – GNL3L – Oligo | **TCGAG**-AATCCCAGCATTTTGGGAG**GCCG** |
| | | AGGCAGG**CGGATCA**CTTGAGGT |
| 9. | Reverse – Sal1 - WT – GNL3L – Oligo | **TCGAC**-ACCTCAAGTGATCCGCCTG |
| | | CCTCGGCCTCCCAAAATGCTGGGATT |
| 10. | Forward – Xho1 - Mut – GNL3L – Oligo | **TCGAG-**AATCCCAGCATTTTGGGAG**GCCG** |
| | | AGGCAGG**AGTATGA**CTTGAGGT |

**Table S2.** List of primers for cloning.

**pSBInducer, shRNA oligos (The mature siRNA/miRNA in the template oligos is underlined.**

miR30PCRXhoI CAGAAGGCTCGAGAAGGTATATTGCTGTTGACAGTGAGCG

miR30PCREcoRI CTAAAGTAGCCCCTTGAATTCCGAGGCAGTAGGCA

**miR-4454**

TGCTGTTGACAGTGAGCGCGAGGGGGAAAGTTCTATAGTCTAGTGAAGCCACAGATGTAGGATCCGAGTCACGGCACCATGCCTACTGCCTCGGA

**miR-4792**

TGCTGTTGACAGTGAGCGCGAGGGGGAAAGTTCTATAGTCTAGTGAAGCCACAGATGTACGGTGAGCGCTCGCTGGCTGCCTACTGCCTCGGA

**siRNA_Scramble:**

TGCTGTTGACAGTGAGCGCAGTGTCAGGGCTAGATACGACTAGTGAAGCCACAGATGTAGTCGTATCTAGCCCTGACACTATGCCTACTGCCTCGGA

**Table S3.** Antibody list.

| Antibody Name | Provider | Cat.log Number |
|---|---|---|
| GNL3L | Abcam | ab94862 |
| p53 (1C12) | Cell Signaling | #2524 |
| p-p53 | Cell Signaling | #9284 |
| MDM2 (SMP14) | Santa Cruz | sc-965 |
| NFkB | Santa Cruz | sc-109 |
| pNFkB (Ser536) (93H1) | Cell Signaling | #3033L |
| CD133 | Proteintech | 184700 |
| Cyclin D | Santa Cruz | sc-246 |
| Twist (2C1a) | Santa Cruz | sc-81417 |
| Snai 1 (T-18) | Santa Cruz | sc-10433 |
| Beta-Catenin | Santa Cruz | sc-1496 |
| IKKalpha/beta | Santa Cruz | sc-7607 |
| p27 (F-8) | Santa Cruz | sc1641 |
| LC3B | Cell Signaling | #2775 |
| EGFR | Cell Signaling | #2232 |
| p-EGFR | Abcam | ab5644 |
| Beta-Actin | Santa Cruz | sc-47778 |

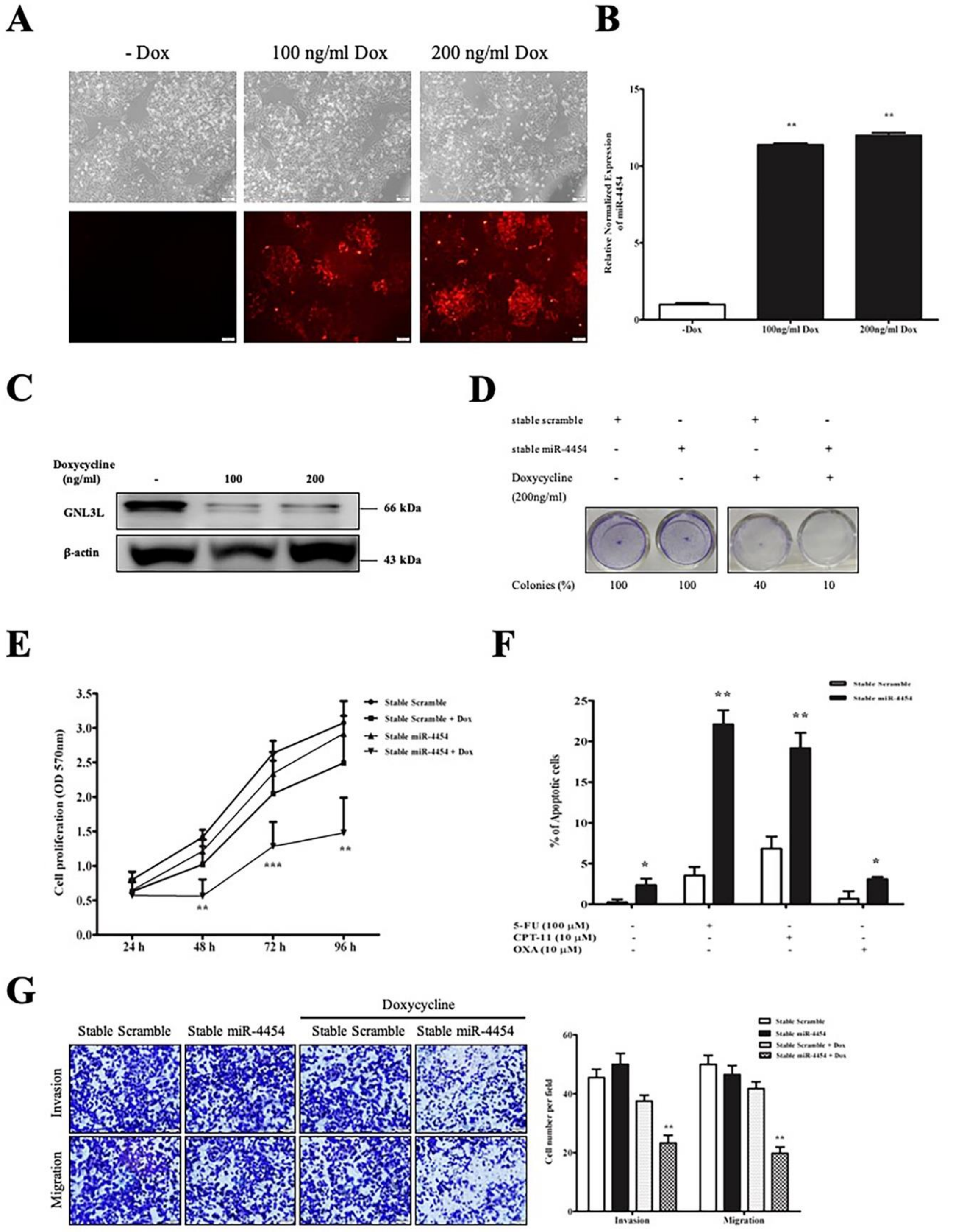
A
- Dox
100 ng/ml Dox
200 ng/ml Dox
B
Relative Normalized Expression of miR-4454
15
10
5
0
**
**
-Dox
100ng/ml Dox
200ng/ml Dox
C
Doxycycline (ng/ml)
-
100
200
GNL3L
β-actin
66 kDa
43 kDa
D
stable scramble + - + -
stable miR-4454 - + - +
Doxycycline (200ng/ml) - - + +
Colonies (%) 100 100 40 10
E
Cell proliferation (OD 570nm)
3.5
3.0
2.5
2.0
1.5
1.0
0.5
0.0
24 h
48 h
72 h
96 h
**
***
**
Stable Scramble
Stable Scramble + Dox
Stable miR-4454
Stable miR-4454 + Dox
F
% of Apoptotic cells
25
20
15
10
5
0
*
**
**
*
Stable Scramble
Stable miR-4454
5-FU (100 μM) - + - -
CPT-11 (10 μM) - - + -
OXA (10 μM) - - - +
G
Doxycycline
Stable Scramble
Stable miR-4454
Stable Scramble
Stable miR-4454
Invasion
Migration
Cell number per field
60
40
20
0
**
**
Invasion
Migration
Stable Scramble
Stable miR-4454
Stable Scramble + Dox
Stable miR-4454 + Dox

# H

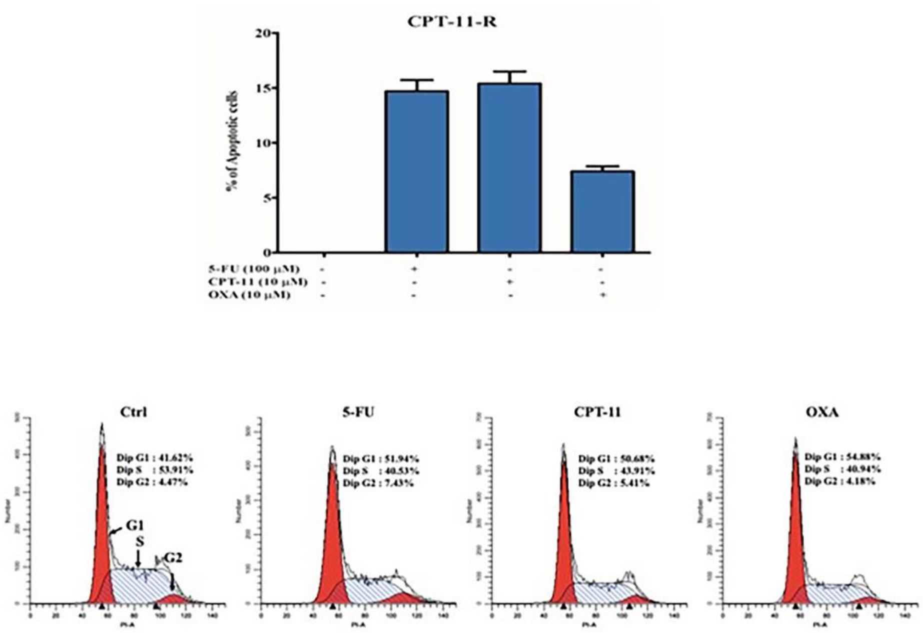


**Figure S1.** Figure depicting single clone selection. (**A**) Doxycycline induction and expression of RFP at different concentrations at 72 h. (**B**) Functional proof that Dox induction leads to overexpression miR-4454 in the CPT-11-R cells. (**C**) Functional proof that Dox-induced miR-4454 overexpression in cells with reduced GNL3L protein expression levels. (**D**) Colony formation assay. (**E**) Cell proliferation assay. (**F**) Analysis of apoptosis after first-line treatment in CPT-11-R scramble or miR-4454-overexpressing cells. (**G**) Invasion/migration assay (right and left). (**H**) CPT-11-R cross-resistance to other anti-cancer drugs (5-FU, Oxaliplatin) through Annexin V apoptosis and cell cycle state.
